# Exaggerated Cardiac Contractile Response to Hypoxia in Adults Born Preterm

**DOI:** 10.3390/jcm10061166

**Published:** 2021-03-10

**Authors:** Gregory P. Barton, Philip A. Corrado, Christopher J. Francois, Naomi C. Chesler, Marlowe W. Eldridge, Oliver Wieben, Kara N. Goss

**Affiliations:** 1 Department of Medical Physics, University of Wisconsin-Madison School of Medicine and Public Health, Madison, WI 53792, USA; gregory.barton@utsouthwestern.edu (G.P.B.); pcorrado2@wisc.edu (P.A.C.); owieben@wisc.edu (O.W.); 2 Department of Pediatrics, University of Wisconsin-Madison School of Medicine and Public Health, Madison, WI 53792, USA; nchesler@uci.edu (N.C.C.); meldridge@pediatrics.wisc.edu (M.W.E.); 3 Department of Internal Medicine, University of Texas Southwestern Medical Center, Dallas, TX 75390, USA; 4 Department of Radiology, University of Wisconsin-Madison School of Medicine and Public Health, Madison, WI 53792, USA; Francois.Christopher@Mayo.edu; 5 Department of Biomedical Engineering, University of Wisconsin-Madison School of Medicine and Public Health, Madison, WI 53792, USA; 6 Department of Medicine, University of Wisconsin-Madison School of Medicine and Public Health, Madison, WI 53792, USA

**Keywords:** cardiac function, contractile response, hypoxia, prematurity

## Abstract

Individuals born prematurely have smaller hearts, cardiac limitations to exercise, and increased overall cardiometabolic risk. The cardiac effects of acute hypoxia exposure as another physiologic stressor remain under explored. The purpose of this study was to determine the effects of hypoxia on ventricular function in adults born preterm. Adults born moderately to extremely preterm (≤32 weeks gestation or <1500 g, *N* = 32) and born at term (*N* = 18) underwent cardiac magnetic resonance imaging under normoxic (21% O_2_) and hypoxic (12% O_2_) conditions to assess cardiovascular function. In normoxia, cardiac function parameters were similar between groups. During hypoxia, the right ventricular (RV) contractile response was significantly greater in participants born premature, demonstrated by greater increases in RV ejection fraction (EF) (*p* = 0.002), ventricular-vascular coupling (VVC) (*p* = 0.004), and strain (*p* < 0.0001) measures compared to term-born participants, respectively. Left ventricular contractile reserve was similar to term-born participants. Adults born preterm exhibit an exaggerated contractile response to acute hypoxia, particularly in the RV. This suggests that adults born preterm may have contractile reserve, despite the lack of volume reserve identified in previous exercise studies. However, this exaggerated and hyper-adapted response may also increase their risk for late RV failure.

## 1. Introduction

Premature birth is defined as birth <37 weeks gestation and accounts for approximately 10% of all births worldwide [1]. Due to improvements in neonatal care in the last three decades, individuals born extremely preterm are surviving and thus account for a growing percentage of the current young adult population. Importantly, adults with a history of prematurity are at increased risk for obstructive lung disease, pulmonary and systemic vascular disease, sleep-disordered breathing, and metabolic diseases such as diabetes and metabolic syndrome [2,3,4]. As a result of these multi-system long-term effects, the National Institute of Health (NIH) now recommends that prematurity be considered a chronic medical condition [2,3].

Recent studies in adults born preterm demonstrate a smaller heart with reduced biventricular chamber size [5,6,7,8,9]. Although left ventricular (LV) function appears preserved, right ventricular (RV) function has been reported as hypercontractile in one study [5] and hypocontractile in others [7,9]. Whether this represents distinct adult cardiac phenotypes after preterm birth, or rather different time points in the transition from adaptive to maladaptive cardiac remodeling, remains to be determined. However, several studies demonstrate impaired cardiac reserve to physiologic stress, particularly exercise. Both adolescents and adults born preterm have a reduced ability to augment stroke volume during exercise, leading to reduced exercise tolerance overall [10,11]. Some studies suggest this may be exaggerated by early right ventricular–pulmonary vascular uncoupling due to underlying preclinical pulmonary hypertension in adults born preterm [12,13].

Although these studies suggest cardiac limitation to physiologic stress in the form of exercise, few studies have evaluated the cardiac response to hypoxia in adults born preterm, which may be particularly relevant given the increased incidence of sleep-disordered breathing after preterm birth [4]. Therefore, the purpose of this study was to determine the cardiac response to acute hypoxia exposure in young adults born preterm. We utilized cardiac magnetic resonance imaging (MRI), which has become the reference standard to quantify cardiac function with specific advantages for assessing RV function [14,15,16], obtaining imaging data during both normoxia and hypoxia. We hypothesized that adults born preterm would have a reduced cardiac adaptation to acute hypoxic stress. Further, due to hypoxic pulmonary vasoconstriction causing greater afterload changes to the RV, we hypothesized that hypoxia would unmask or accentuate reduced RV function in individuals with a history of prematurity, and that this attenuated ventricular response would be greater in individuals with a neonatal diagnosis of bronchopulmonary dysplasia (BPD).

## 2. Materials and Methods

### 2.1. Participants and Study Design

Young adult participants born moderately to extremely premature (*N* = 32) were recruited from the Newborn Lung Project, a cohort of infants born with very low birth weight (≤1500 g) between 1988 and 1991 in Wisconsin and Iowa and followed prospectively at the University of Wisconsin-Madison [17,18,19], or from the general public with confirmation of birth history from neonatal records (birth weight ≤1500 g or gestational age ≤32 weeks). Similarly aged term-born participants (*N* = 18) were recruited from the local population. All participants were free of current cardiovascular or respiratory illness, diabetes (fasting blood glucose <126 mg/dL), and nonsmokers. For preterm participants with a history of BPD, BPD was defined clinically by the use of supplemental oxygen at 36 weeks’ postmenstrual age. All participants were informed of the purpose and risks of the study and provided written informed consent in accordance with the standards set by the Declaration of Helsinki. The protocols were approved by the Institutional Review Board (2017-0238) at the University of Wisconsin-Madison. The study was registered at clinicaltrial.gov (NCT03245723).

**Figure 1:**
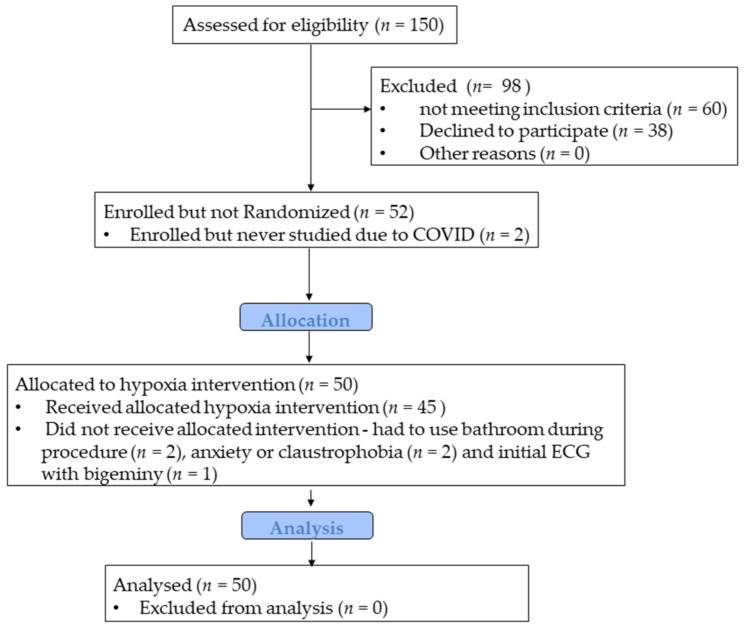
Schematic showing participant flow through the study.

Prior to the start of the MRI study, all participants underwent a resting ECG screening exam (to ensure no arrhythmias that would preclude hypoxia exposure) and baseline anthropometric measurements. The overall design of the MRI study included participants breathing normoxic air (21% O_2_) for 25 min followed by a hypoxic gas mixture (12% O_2_) for 45 min in a serial fashion while inside the MRI bore. Both normoxic and hypoxic air was delivered to participants wearing a face mask with a two-way valve attached to a hose which was connected to normoxic and hypoxic air bags inside the MRI room. Hypoxic MRI began after a 25 min wash-in of hypoxic gas. Four preterm and one term-born participants did not perform hypoxia due to claustrophobia from wearing the mask (*N* = 2), having to abruptly stop the MRI exam to use the restroom (*N* = 2), or initial ECG with bigeminy precluding hypoxia exposure (*N* = 1, preterm) (Figure 1).

### 2.2. Cardiovascular Magnetic Resonance Imaging (MRI) Acquisition

Cardiac MRI was serially acquired while participants were breathing normoxic air and hypoxic gas using a 3.0 T PET-MRI scanner (GE Signa PET/MR Discovery 750W, GE Healthcare, Waukesha, WI, USA). To characterize cardiac function, a prospectively ECG-gated, multi-slice balanced steady-state free precession (bSSFP) short-axis sequence was acquired during an end-expiratory breath hold with field of view = 35 × 35 cm; spatial resolution = 1.4 × 1.4 mm; slice thickness = 7 mm; number of slices = 17; breath hold length/slice acquired = 13 s; reconstructed cardiac phases = 20. Short-axis data from the normoxic MRI acquisition were published previously [5]. In order to probe for differences in vascular function, 2D phase contrast (PC) imaging was used to assess pulmonary artery (PA) and ascending aortic (Ao) diameter, mean velocity, and relative area change (RAC). 2D PC images were ECG-gated and acquired during an end-expiratory breath hold with velocity encoding setting (VENC) = 180 cm/s; spatial resolution = 1.4 × 1.4 mm; flip angle = 20°; TR/TE (5.584/3.376 ms); slice thickness = 7 mm; breath hold length = 8 s; reconstructed cardiac phases = 20.

### 2.3. Image Analysis

Analysis of volumetric cardiac images was performed by an analyst from the University of Wisconsin-Madison Medical Imaging Research Support-Image Analysis (MIRS-IA) core lab blinded to birth history, using commercially available software (cvi42, version 5.6.6 (859), Circle Cardiovascular Imaging, Inc., Calgary, AB, Canada). RV and LV end-diastolic volume (EDV), end-systolic volume (ESV), stroke volume (SV), and cardiac output (CO) were calculated from short axis cine bSSFP series. Ventricular volumes were indexed to body surface area. Three slices from the left and right ventricle (apex, mid, and base) short-axis cine MR images were used to analyze circumferential and radial dimensions and strain, including average peak strain, systolic, and diastolic strain rate, using feature tracking via a commercially available software (Segment, version 2.2 R6423 strain analysis module; Medviso, Lund, Sweden, http://segment.heiberg.se) [20]. PA and Ao diameter, peak mean velocity, and relative area change were calculated from the 2D PC images, with RAC calculated by: (maximum vessel area − minimum vessel area)/maximum vessel area [21].

### 2.4. Statistical Analysis

Data were initially grouped by birth status (preterm, term). Baseline anthropometric, normoxic, and hypoxic MRI were compared across groups using a two-sample Student’s *t*-test. To evaluate whether there was an interaction between birth status and the effect of hypoxia exposure on cardiac MR measurements, we performed a two-way mixed effects model. In order to address our secondary aim of whether a history of BPD would affect ventricular responses to hypoxia to a greater extent than the preterm born participants without BPD, we utilized a two-way mixed effects model to determine the main effect of birth status (preterm, preterm_BPD, term), main effect of hypoxia, and the interaction between birth status and hypoxia on all MRI measurements. Significance level was determined a priori at the 0.05 level and all tests were 2-tailed. Data were presented as mean ± standard deviation, unless otherwise noted. Statistical analyses were performed using Prism Graphpad (Version 8, GraphPad Software Inc., La Jolla, CA, USA).

## 3. Results

### 3.1. Baseline Characteristics

Adults born preterm were of similar age and sex distribution to the term-born control participants (Table 1). Preterm participants were significantly shorter and had a non-significantly higher body mass index (BMI). Preterm participants had an average gestational age of 29.2 weeks, with roughly one-third having a neonatal history of BPD or a patent ductus arteriosus (PDA, including 8 treated pharmacologically and 3 treated surgically). Hemoglobin was higher in the preterm participants compared to term participants (15.8 ± 2.3 vs. 14.2 ± 1.7, *p* = 0.01) as reported previously [22]. Lung function including forced vital capacity and forced expiratory volume in 1 s was similar between groups, as previously reported [22].

### 3.2. Cardiovascular Responses to Normoxia

In normoxic conditions, systolic (SBP) and diastolic blood pressure (DBP), heart rate, cardiac index (CI), and stroke volume index (SVI) were similar between term and preterm participants (Table 2). As reported previously, biventricular volumes including end-diastolic volume index (EDVI) and end-systolic volume index (ESVI) were smaller in the preterm participants [5] (Table 2). Biventricular ejection fraction (EF) and ventricular-vascular coupling (SV/ESV) were higher in preterm participants, although not reaching statistical significance. With respect to strain, there were no differences in peak strain, systolic, or diastolic strain rate in the LV (Table 3). However, preterm participants exhibited increased RV peak circumferential strain and RV circumferential systolic strain rate as compared to term participants.

We utilized 2D PC MRI to characterize the cross-sectional area and flow in the great vessels throughout the cardiac cycle. There were no differences between groups with respect to PA and Ao diameter, Ao:PA diameter ratio, Ao mean velocity, and Ao RAC (Table 4). However, pulmonary artery RAC and PA mean velocity were significantly lower in the preterm group, suggesting a stiffer PA and underlying pulmonary hypertension.

### 3.3. Cardiovascular Responses to Hypoxia

**Figure 2:**
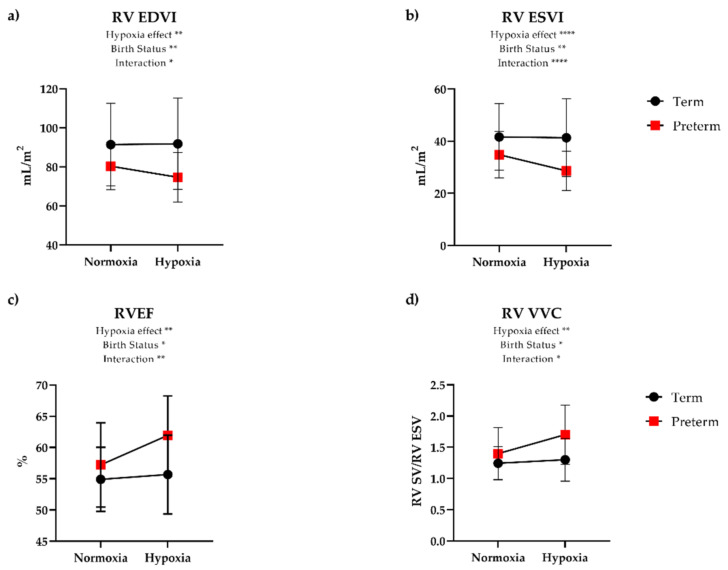
Right ventricular (RV) functional response is exaggerated in adults born preterm. There are significant decreases in (**a**) RV end-diastolic (EDVI) (*p* = 0.01) and (**b**) end-systolic volume index (ESVI) (*p* ≤ 0.0001) in preterm individuals, while term born-individuals do not see any decreases in RV EDVI or ESVI. Interestingly, individuals born preterm exhibit a robust contractile response to hypoxia as observed by dramatic increases in (**c**) RV ejection fraction (EF) (*p* = 0.009) and (**d**) RV ventricular—vascular coupling (VVC) (*p* = 0.01), while no changes were observed in term-born individuals. Data represent mean ± SD. * *p* < 0.05, ** *p* < 0.01, **** *p* < 0.0001.

**Figure 3:**
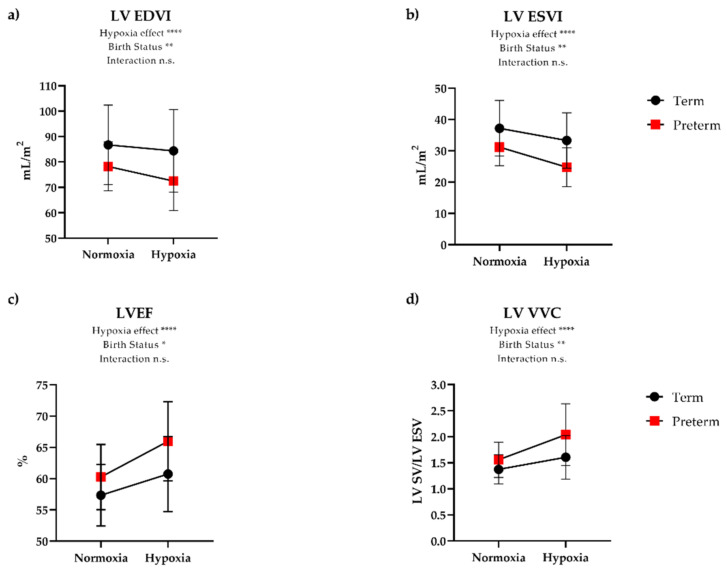
Left ventricular (LV) response to hypoxia. There was a main effect of hypoxia and birth status for all LV variables. Namely, there was a 3% and 8% reduction in (**a**) LV EDVI from normoxia to hypoxia in term and preterm participants, respectively (*p* = 0.03). Acute hypoxia resulted in a 10% and 18% reduction in (**b**) LV ESVI in term and preterm, respectively (*p* = 0.03). There were similar relative changes in (**c**) LVEF from normoxia to hypoxia between term (5%) and preterm (8%) participants. LV ventricular vascular coupling (VVC) (**d**) improved in both groups as demonstrated by an increase in preterm (28%) and term-born (15%) participants, although this did not reach statistical significance (*p* = 0.14). Data represent mean ± SD. * *p* < 0.05, ** *p* < 0.01, **** *p* < 0.0001, n.s. = not significant (*p* > 0.05).

**Figure 4:**
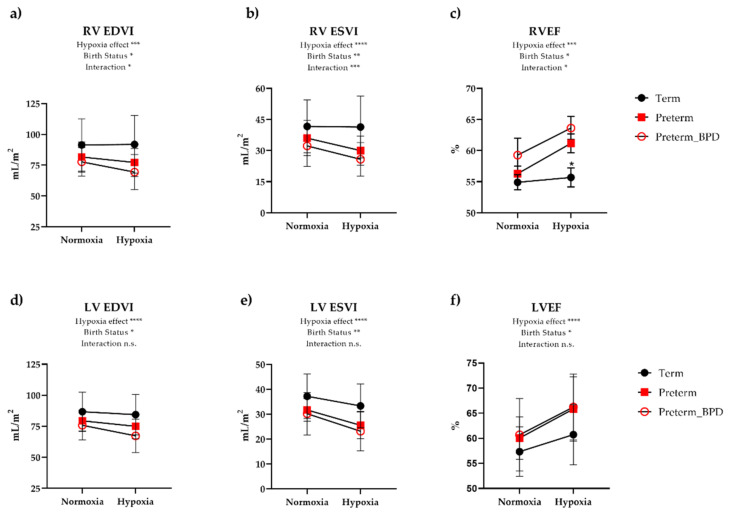
Biventricular function stratified by preterm with or without a history of bronchopulmonary dysplasia (BPD). There are significant decreases in (**a**–**c**) right ventricular (RV) end-diastolic index (EDVI) (*p* = 0.029) and end-systolic volume index (ESVI) (*p* = 0.0005) in preterm individuals with or without a diagnosis of BPD, while term born-individuals do not see any decreases in RV EDVI or ESVI. Interestingly, individuals born preterm with or without BPD exhibit a robust contractile response to hypoxia as observed by dramatic increases in RV ejection fraction (EF) (*p* = 0.026), while no changes were observed in term-born individuals. There was a main effect of hypoxia for all LV variables (**d**–**f**). The LV functional responses between groups were quantitatively different, although not statistically significant. Namely, there was a 3%, 6%, and 11% reduction in LV EDVI from normoxia to hypoxia in term, preterm, and preterm with BPD, respectively. Acute hypoxia resulted in a 10%, 18%, and 18% reduction in LV ESVI in term, preterm, and preterm with BPD, respectively. There were similar relative changes in LVEF from normoxia to hypoxia in all three groups (range 6–8% increase). In order to determine if there were differences between those with and without a history of BPD, we utilized an unpaired *t*-test and found no differences (*p* > 0.05) for all of the RV and LV measures. Data represent mean ± SD. * *p* < 0.05, ** *p* < 0.01, *** *p* < 0.001, **** *p* < 0.0001, n.s. = not significant (*p* > 0.05).

During inhalation of 12% oxygen, both groups had a similar degree of desaturation when averaged over the hypoxia exposure (mean 83% in term vs. 82% in preterm), and there was no significant change in SBP or DBP in either group. There was an increase in HR in both groups, although preterm participants exhibited an 18% increase in HR while term participants had a 9% increase (Table 2). Although CI increased similarly in both groups during hypoxia, there was an exaggerated contractile response to hypoxia in the preterm participants. Somewhat surprisingly, this response was greater in the RV (Figure 2) than the LV (Figure 3). Namely, RV EDVI and ESVI decreased (8.0% and 17%, respectively) during hypoxia in preterm participants, while term participants had minimal change (0.3% and 1.0% decrease, respectively). Despite evidence of a reduction in RV preload (i.e., EDVI) and presumed higher RV afterload in preterm participants based on lower PA RAC, RV SVI was maintained in hypoxia by augmenting contractility, as evidenced by increased RV EF (7.5% increase), RV VVC (21% increase), and RV peak circumferential strain (8% increase). These contractile changes were similar in preterm participants with and without a history of BPD (Figure 4). Further, we did not see any differences in preterm participants with or without a patent ductus arteriosus. In contrast, participants born at term showed minimal changes to RVEF (0.6%), RV VVC (3.0%), and RV peak circumferential strain (−3.0%). Linear regression analysis revealed that the functional response to hypoxia in the LV EF and RV EF were more coupled in preterm group (R^2^ = 0.45, *p* < 0.0001) and less so in the term-born participants (R^2^ = 0.19, *p* = 0.08).

**Figure 5:**
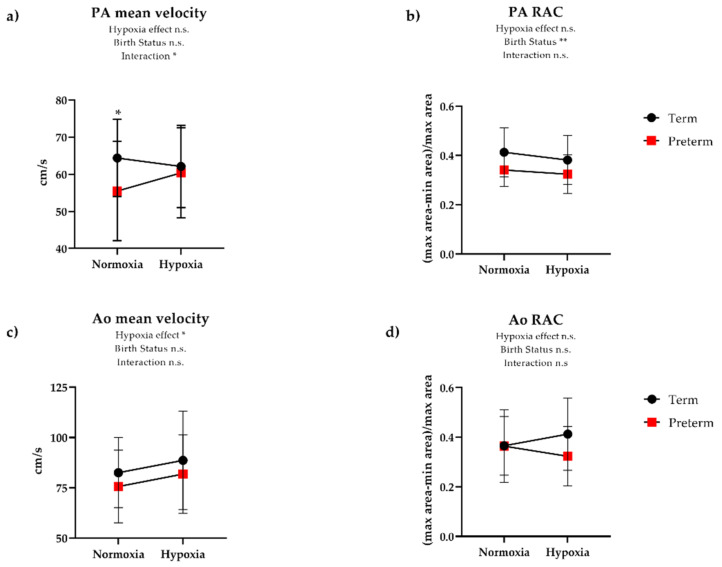
Hypoxic mediated changes in the (**a**,**b**) pulmonary artery (PA) and (**c**,**d**) aorta (Ao) by 2D phase contrast (PC) imaging. Individuals born preterm exhibit increased PA mean velocity during hypoxia, while there is a subtle reduction in PA mean velocity in term-born participants (*p* = 0.03). The increased PA mean velocity observed in preterm participants is likely a result of the increased RV contractile response to hypoxia. Overall, individuals born preterm display reduced baseline PA relative area change (RAC) which suggests a stiffer PA compared to term-born participants. There is a subtle reduction to PA relative area change (RAC) during hypoxia in term and preterm participants, suggesting increased PA stiffening. Overall, there were no significant changes in Ao mean velocity or RAC. Data represent mean ± SD. * *p* < 0.05, ** *p* < 0.01, n.s. = not significant (*p* > 0.05).

Term and preterm participants exhibited similar changes in Ao mean velocity and RAC during hypoxia. However, there was a similar reduction in PA RAC during hypoxia in both term and preterm groups (Table 4, Figure 5). In hypoxia, PA mean velocity increased in preterm participants, whereas term participants showed no change. There were no significant differences in the PA and Ao mean velocity or RAC response to hypoxia between preterm participants with and without a history of BPD.

Due to the slightly unbalanced nature in our groups (higher BMI and more females in the preterm group), we performed additional analysis and found there was no significant effect of biological sex or BMI on the cardiac functional response to hypoxia.

## 4. Discussion

The purpose of this study was to determine ventricular adaptation to acute hypoxia exposure in individuals with a history of premature birth. We initially hypothesized that adults born preterm would have a reduced cardiac adaptation to acute hypoxic stress, with greater impairment in the RV response due to expected hypoxic pulmonary vasoconstriction and elevated afterload [23,24]. Contrary to our hypotheses, we found that participants born premature with normal resting cardiac function exhibited an exaggerated contractile response to hypoxia, particularly in the RV, regardless of neonatal BPD status. The LV in preterm participants adapted in similar fashion to term-born controls during hypoxia. Collectively, young adults born premature exhibit a differential RV functional response to acute hypoxia demonstrated by a hypercontractile phenotype during hypoxic stress.

Acute hypoxia exposure in healthy volunteers results in increased heart rate, cardiac output, pulmonary vascular pressure, LV contractile function, and RV systolic pressure [12,23,24,25], similar to what we reported here in the term-born control group. In previous studies in the preterm population, acute hypoxia exposure in infants with BPD resulted in an exaggerated pulmonary vasoconstrictor response [26]. However, 8–12-year-old children born preterm with and without BPD demonstrated similar cardiac and pulmonary vascular responses to acute hypoxia exposure as assessed by echocardiography [27]. Among adults previously recruited from the Newborn Lung Project, mean PA pressures were higher both at rest and during hypoxia, though there did not appear to be a greater hypoxic pulmonary vasoconstrictor response in those preterm [12]. Given that we identified early RV-pulmonary vascular uncoupling in a prior study, [13] we hypothesized that further hypoxic pulmonary vasoconstriction would worsen the RV functional response to hypoxia, and this attenuated RV response to hypoxia would be further exacerbated by a history of BPD.

Surprisingly, we identified an exaggerated contractile reserve in the RV during hypoxia in this cohort with preserved baseline RV function. The RV response to hypoxia in preterm-born individuals manifested as a 7.5% increase in RVEF, identical to the 8% increase in the PA mean velocity, suggesting the change in PA mean velocity was likely the result of augmented RV contractility in preterm participants. Additionally, there was a 21% increase in RV VVC despite a presumed higher RV afterload in hypoxia [12], which demonstrates a hypercontractile adaptation given a similar degree of hypoxia compared to term-born participants. Hypoxic pulmonary vasoconstriction appears to be similar as we demonstrated here that PA RAC decreases (i.e., becomes stiffer), and previously that mPAP increases [12], in similar fashion from normoxia to hypoxia in preterm and term participants, respectively. Taken together, the augmented RV contractility observed in preterm participants in this study appears to be out of proportion to the increase in RV afterload based on findings of similar hypoxic pulmonary vasoconstrictor response between preterm and term-born participants. Furthermore, we did not see significant differences in preterm participants with or without a history of BPD. However, the preterm participants with BPD in this study (*N* = 10) had mild to moderate BPD and none required respiratory support beyond low-flow nasal cannula at NICU discharge. Thus, whether the same results would be identified in long-term follow-up studies of more severe BPD remain to be seen. Further, there was no clear effect of having a PDA on the response to acute hypoxia.

The use of physiological stressors such as exercise and hypoxia have been important for delineating functional differences between adults with or without a history of premature birth. Previous work has shown that individuals born premature exhibit reduced exercise capacity [12,28,29,30,31,32]. Notably, our group has shown that young adults born preterm have a blunted SV response to exercise which was related to attenuated RV stroke work pointing to a cardiac limitation to exercise in this population [12]. Others demonstrate impaired LV reserve to exercise as well [10,11]. Given our findings of exaggerated RV contractile response to hypoxia, it is important to consider the inherent differences in the cardiac response to hypoxia and exercise. Hypoxia results in a much smaller increase in cardiac output relative to exercise. The greater effect appears to be increased RV afterload due to hypoxic pulmonary vasoconstriction, and presents a modest pressure challenge to the RV. In contrast, exercise elicits a large increase in cardiac output and is considered to also be a volume challenge on the myocardium. Taken together, it appears that the preterm RV is hyperadaptive to a pressure challenge, but maladaptive to a volume challenge. This may be primarily driven by the smaller biventricular chamber size in the preterm heart [5], such that there is limited volume reserve for augmenting cardiac output.

Intriguingly, previous work in rats using postnatal hyperoxia, a model of preterm birth in humans, also showed increased right ventricular and exercise performance after two weeks of hypoxia [33], though the mechanisms for this favorable adaptation remain to be elucidated and biochemical adaptations that favor hypoxia should be evaluated in future studies. Unfortunately, this compensation does not persist, as rats exposed to postnatal hyperoxia still developed late RV failure and RV-pulmonary vascular uncoupling associated with impaired mitochondrial biogenesis and function with progressive accumulation of mitochondrial DNA damage, suggesting these early hyperadaptations do not prevent long term failure [34,35].

Hypoxia is a particularly relevant physiologic stressor for the preterm population, as individuals born premature are at increased risk of developing sleep-disordered breathing and intermittent repeated bouts of hypoxia [4]. The hypercontractile state of the RV in response to hypoxia demonstrated in this study would result in marked increases in myocardial oxygen demand during hypoxia. Translating this hypercontractile phenotype to the broader population of individuals born preterm who are at increased risk of sleep-disordered breathing could result in repeated bouts of a hypercontractile state, which would chronically stress the energetic demands of the mitochondria. Combining a history of prematurity with sleep-disordered breathing has potential to be deleterious to the preterm heart.

Previous work in young adults from the Newborn Lung Project cohort showed that individuals with a history of prematurity exhibit abnormal ventilatory response to hypoxia [36]. The authors of this article postulated that the altered ventilatory response to hypoxia in preterm subjects is possibly due to perturbed chemoreceptor development [37], and chemoreceptors are known to regulate autonomic nervous system function [38]. Although speculative in nature, taken together with the hypercontractile RV response to hypoxia relative to the term-born RV observed herein and previous work demonstrating altered ventilatory response to hypoxia, this potentially points to perturbed chemoreceptor response to hypoxia and altered autonomic nervous system function. Previous work using indirect methods to determine autonomic function have shown perturbations in adolescents [39] and adults born preterm [29]. Future work utilizing direct methods to determine autonomic outflow and the relationship to cardiac and pulmonary function will be important to determine the role the autonomic nervous system has on altered physiological responses to stress.

Study strengths included the use of cardiac MRI to study biventricular function using a relevant physiologic stress in a well characterized population of adults born moderately to extremely preterm. Limitations to this study are sample size, including only 10 out of the 32 preterm participants who had a prior diagnosis of BPD, with all of them meeting criteria for mild to moderate BPD under current definitions. Therefore, we may not have fully captured a differential response to hypoxia between preterm participants with or without BPD. Further, our participants had normal baseline cardiac function, and thus it remains unclear if individuals with baseline reduced RV function have contractile reserve. Another limitation to this study is that we did not acquire long-axis cine images during hypoxia, which did not permit the ability to probe changes in RV longitudinal strain. It is known that ~70–80% of systolic function in the RV is the result of longitudinal contraction with the remaining 20–30% of systolic function due to transverse or circumferential contraction [40]. Therefore, this limited our ability to determine the extent of an exaggerated RV response to hypoxia in preterm born adults. Of additional importance was the challenge of comparing strain values across studies due to the inherent differences in the outputs of analysis software. Multicenter trials using the same cardiac imaging acquisition and analysis tools would help alleviate these challenges. Future studies with larger sample sizes would help to address the question of cardiac phenotypes stratified by perinatal and neonatal factors that may be important to predicting functional response to stress. Furthermore, the role of potential biochemical or metabolic adaptations to promote RV hypercontractility, as well as the potential for exaggerated autonomic responses to hypoxia, should be evaluated in future preterm studies.

## 5. Conclusions

In summary, adults with a history of premature birth and normal baseline cardiac function exhibit an exaggerated contractile response to hypoxia, particularly in the RV, which was unaffected by neonatal history of BPD or PDA. This suggests that while the preterm heart maintains contractile reserve, hyper-adapted responses to hypoxia including sleep-disordered breathing or altitude could be deleterious in the long term and increase risk for late RV failure. Additional studies assessing both neonatal and adult factors contributing to abnormal cardiopulmonary stress testing are warranted, and future studies should pay careful attention to both the right and left ventricular responses.

## Figures and Tables

**Table 1:** Baseline demographics (mean ± SD).

	Term	Preterm	*p*-Value
**Baseline Adult Characteristics**			
Number, Participants (*N*)	18	32	
Current age, years	25.4 (4.0)	26.1 (4.2)	0.58
Female sex	10 (56%)	23 (72%)	0.25
Race, white	17 (94%)	30 (94%)	0.92
Height (inches)	68.1 (3.4)	65.6 (2.8)	<0.01
Weight (kg)	69.9 (9.6)	74.5 (24.1)	0.44
Body mass index (kg/m^2^)	23.3 (1.9)	26.7 (8.5)	0.10
Body surface area (m^2^)	1.83 (0.17)	1.84 (0.29)	0.91
**Neonatal Characteristics**			
Gestational age (weeks)	39.8 (1.0)	29.2 (2.5)	<0.01
Birth weight (g)	3397 (572)	1225 (397)	<0.01
Bronchopulmonary dysplasia	n/a	10 (32%)	
Days on assisted ventilation	n/a	12.4 (14.0)	
Patent ductus arteriosus	n/a	12 (38%)	

**Table 2:** Cardiac hemodynamic response to hypoxic stress (mean ± SD).

	Normoxia			Hypoxia			
Variable	Term (*N* = 18)	Preterm (*N* = 32)	(Normoxia) *p*	Term (*N* = 17)	Preterm (*N* = 28)	(Hypoxia) *p*	(Interaction) *p*
SBP (mmHg)	123 (11)	126 (18)	0.50	126 (18)	125 (17)	0.78	0.24
DBP (mmHg)	72 (7)	75 (10)	0.23	74 (7)	74 (11)	0.94	0.15
SpO_2_ (%)	98.1 (1.1)	98.0 (1.1)	0.87	83 (5)	82 (8)	0.51	0.35
HR (b/min)	67 (13)	70 (11)	0.47	72 (13)	81 (15)	0.05	0.12
LV EF (%)	57 (5)	60 (5)	0.06	61 (6)	66 (6)	<0.01	0.25
LV CI (L/min/m^2^)	3.29 (0.78)	3.26 (0.59)	0.89	3.60 (0.61)	3.81 (0.76)	0.34	0.30
LV EDVI (mL/m^2^)	86.7 (15.6)	78.2 (9.6)	0.02	84.4 (16.3)	72.5 (11.6)	<0.01	0.06
LV ESVI (mL/m^2^)	37.2 (8.9)	31.2 (6.0)	<0.01	33.3 (8.8)	24.8 (6.2)	<0.01	0.11
LV SVI (mL/m^2^)	49.5 (8.9)	47.0 (6.6)	0.26	51.1 (10.5)	47.7 (8.5)	0.25	0.54
LV VVC (SV/ESV)	1.37 (0.28)	1.56 (0.34)	0.06	1.61 (0.42)	2.04 (0.59)	0.01	0.12
RV EF (%)	55 (5)	57 (7)	0.21	56 (6)	62 (6)	<0.01	0.01
RV CI (mL/min/m^2^)	3.28 (0.70)	3.15 (0.54)	0.48	3.55 (0.60)	3.67 (0.72)	0.56	0.23
RV EDVI (mL/m^2^)	91.4 (21.2)	80.4 (12.0)	0.02	91.8 (23.4)	74.7 (12.6)	<0.01	0.01
RV ESVI (mL/m^2^)	41.6 (12.7)	34.8 (8.9)	0.03	41.3 (14.9)	28.6 (7.5)	<0.01	<0.01
RV SVI (mL/m^2^)	49.8 (10.0)	45.6 (6.5)	0.08	50.5 (11.0)	46.0 (8.1)	0.12	0.84
RV VVC (SV ESV)	1.25 (0.27)	1.40 (0.42)	0.16	1.30 (0.34)	1.70 (0.47)	<0.01	0.01

Abbreviations: SBP = systolic blood pressure, DBP = diastolic blood pressure, SpO_2_ = peripheral oxygen saturation, LV = left ventricle, RV = right ventricle, EDVi = end diastolic volume index, ESVi = end systolic volume index, SVi = stroke volume index, EF = ejection fraction, CI = cardiac index, VVC = ventricular–vascular coupling.

**Table 3:** Cardiac mechanical response to hypoxic stress (mean ± SD).

	Normoxia			Hypoxia			
Variable	Term (*N* = 18)	Preterm (*N* = 32)	(Normoxia) *p*	Term (*N* = 16)	Preterm (*N* = 27)	(Hypoxia) *p*	(Interaction) *p*
LV peak radial strain (%)	23.4 (9.1)	27.0 (9.3)	0.19	25.4 (9.8)	32.8 (10.6)	0.03	0.26
LV radial systolic SR (%/s)	95.2 (41.3)	107.8 (40.8)	0.30	102.8 (43.4)	142.4 (59.9)	0.03	0.06
LV radial diastolic SR (%/s)	−103.4 (52.5)	−118.2 (58.6)	0.38	−128.8 (71.1)	−157.4 (80.3)	0.24	0.50
LV peak circumferential strain (%)	−16.2 (2.7)	−17.9 (3.3)	0.08	−16.7 (3.4)	−19.6 (3.2)	<0.01	0.33
LV circumferential systolic SR (%/s)	−77.2 (15.7)	−81.7 (16.9)	0.36	−83.5 (23.0)	−102.3 (24.1)	0.02	0.04
LV circumferential diastolic SR (%/s)	68.0 (18.7)	70.7 (19.2)	0.63	72.2 (27.3)	88.7 (31.3)	0.09	0.06
RV peak circumferential strain (%)	−8.1 (1.8)	−10.6 (2.6)	<0.01	−8.1 (2.0)	−11.4 (2.2)	<0.01	0.42
RV circumferential systolic SR (%/s)	−39.0 (13.2)	−47.9 (14.1)	0.03	−41.5 (12.2)	−57.3 (16.3)	<0.01	0.06
RV circumferential diastolic SR (%/s)	33.2 (12.8)	40.6 (13.8)	0.07	32.6 (11.6)	40.0 (9.2)	0.03	0.90

**Table 4:** Vascular response to hypoxic stress (mean ± SD).

	Normoxia			Hypoxia			
Variable	Term (*N* = 18)	Preterm (*N* = 32)	(Normoxia) *p*	Term (*N* = 17)	Preterm (*N* = 28)	(Hypoxia) *p*	(Interaction) *p*
Ao mean velocity (cm/s)	83 (17)	76 (18)	0.21	89 (24)	82 (19)	0.32	0.72
Ao diameter (mm)	26.7 (2.6)	26.6 (3.6)	0.86	26.9 (3.1)	26.1 (3.0)	0.39	0.81
Ao RAC	0.37 (0.12)	0.36 (0.15)	0.98	0.41 (0.15)	0.32 (0.12)	0.03	0.10
PA mean velocity (cm/s)	64 (10)	55 (13)	0.02	62 (11)	60 (12)	0.64	0.03
PA diameter (mm)	27.0 (7.5)	29.0 (3.0)	0.19	29.2 (4.2)	28.8 (3.1)	0.69	0.14
PA RAC	0.41 (0.10)	0.34 (0.07)	<0.01	0.38 (0.10)	0.32 (0.08)	0.04	0.74
Ao:PA diameter	0.94 (0.13)	0.92 (0.10)	0.45	0.93 (0.12)	0.91 (0.12)	0.63	0.37

## Data Availability

The data that support the findings of this study are available on request from the corresponding author. The data are not publicly available due to privacy or ethical restrictions.
